# Alkaline Phosphatase Controls Lineage Switching of Mesenchymal Stem Cells by Regulating the LRP6/GSK3β Complex in Hypophosphatasia: Erratum

**DOI:** 10.7150/thno.103686

**Published:** 2024-09-22

**Authors:** Wenjia Liu, Liqiang Zhang, Kun Xuan, Chenghu Hu, Liya Li, Yongjie Zhang, Fang Jin, Yan Jin

**Affiliations:** 1 MS-State Key Laboratory & National Clinical Research Center for Oral Diseases & Shaanxi International Joint Research Center for Oral Diseases, Center for Tissue Engineering, School of Stomatology, Fourth Military Medical University, Xi'an, 710032, China.; 2 Xi'an Institute of Tissue Engineering and Regenerative Medicine, Xi'an, 710032, China.

The authors regret to find an error in the published version of Figure 2E and 3B, where the images were misused. After carefully re-verifying the original data, the authors identified a labeling error that occurred during the image capture process. The corrected version of Figure 2E and 3B appears below. The corrections made in this erratum do not affect the original conclusions. The authors apologize for any inconvenience that the errors may have caused.

## Figures and Tables

**Figure 2E:**
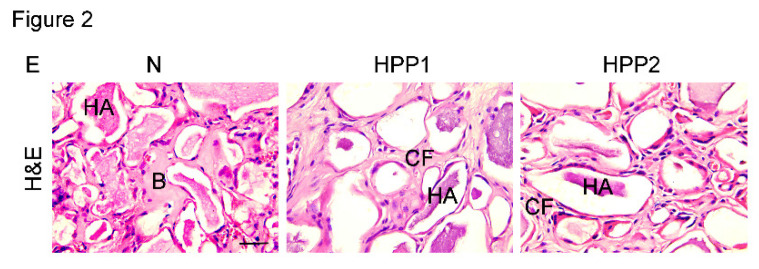
Correct image.

**Figure 3B:**
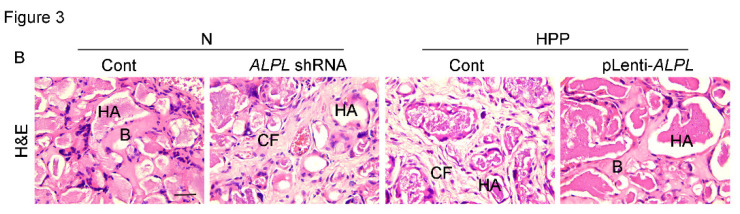
Correct image.

